# Factors associated with health-related quality of life in adults with asthma. A cross-sectional study

**DOI:** 10.1186/2049-6958-7-32

**Published:** 2012-10-02

**Authors:** Francisco-Javier Gonzalez-Barcala, Ramon de la Fuente-Cid, Mónica Tafalla, Javier Nuevo, Francisco Caamaño-Isorna

**Affiliations:** 1 Pneumology Department, Clinic University Hospital, C/Choupana SN 15706, Santiago de Compostela, Spain; 2 Internal Medicine Department, Clinic University Hospital, Santiago de Compostela, Spain; 3 Medical Department, AstraZeneca Farmacéutica, Madrid, Spain; 4 CIBER of Epidemiology and Public Health (CIBERESP), Preventive Medicine Department, University of Santiago de Compostela, Santiago de Compostela, Spain

**Keywords:** Asthma, Quality of life, Risk factors, Adults

## Abstract

**Background:**

The measurement of health-related quality of life (HRQoL) is increasingly recognized as an important endpoint, as a reflection of the effects of the disease from a patient perspective. Our aim was to evaluate the factors determining the HRQoL in patients with asthma, according to the EQ-5D questionnaire.

**Methods:**

Patients were included using multi-stage sampling, from Primary Care clinics from all the Autonomous Communities in Spain. The patients included were: over 18 years-old, with a confirmed diagnosis of asthma for at least one year, and had visited a Health Centre in the previous 2 years. The characteristics of the asthma disease, the adherence to treatment, the socio-demographic variables, the smoking habits, and the asthma control were collected using a questionnaire. The influence of the different variables included in the study on the EQ-5D was evaluated using multivariate logistic regression analysis.

**Results:**

A total of 2,125 patients were finally included (57.7% females, mean age 48 years). The response rate was 95.4%. Some factors showed a considerable detrimental effect on the HRQoL of asthmatics. Advanced age, lower educational level and poor control of asthma are significantly associated with a worse quality of life in all the dimensions assessed by the EQ-5D scale. The baseline severity of the asthma, and having been admitted to hospital are related to a worse quality of life in 5 of the 6 dimensions analyzed.

**Conclusion:**

In our study, we could identify some factors related to quality of life in asthma patients. The most important were advanced age, lower education level, and poor control of the asthma.

## Background

The measurement of health-related quality of life (HRQoL) is increasingly recognized as an important endpoint, a reflection of the effects of the disease from a patient’s perspective. It covers a multidimensional concept, not only associated with the disease itself and the medical actions developed for its management, but also with the physical, social and psychological functioning of the patient [1-5]. Although clinical and physiological parameters are needed to evaluate the disease, these are not sufficient to understand and assess how the patients perceive their state of health [6,7].

It is known that asthma negatively influences the quality of life of the patients who suffer from it, but the determining factors are not fully understood. In fact, the most severe forms of the disease are associated with a worse quality of life, but this relationship is not linear [8,9]. The factors related to this aspect of the disease need to be identified in order to improve the quality of life of the patients [7-9]. Several questionnaires have been developed to assess HRQoL. Some are used for specific diseases, while others are generic [7].

The EuroQol-5D (EQ-5D) questionnaire is a generic tool for measuring HRQoL, applicable in healthy individuals as well as in patients with a specific disease, which seems to have demonstrated validity and reliability in asthma [7]. In this, five health dimensions, stratified into 3 levels, as well as visual analogue scale (VAS) are descriptively evaluated [7].

The EQ-5D is a generic HRQoL questionnaire, and although not specific for asthma, it is considered valid for cross-sectional population studies of patients with this disease [2].

Our aim was to evaluate the factors associated with HRQoL in patients with asthma, from a multidimensional perspective.

## Methods

Patients were included, using multi-stage sampling, from Primary Care clinics from all the Autonomous Communities in Spain. The first sampling unit was the Family Doctors, with 182 doctors being selected. They, in turn, included between 12 and 20 consecutive patients who were seen at their clinics, and who met with the previously defined criteria [10]. The patients were contacted by telephone from the clinic by their own doctor, and the data was collected by personal interview between November 2007 and March 2008.

The patients included were those who were over 18 years-old (adult legal age in Spain), with a confirmed diagnosis of asthma for at least one year according to GINA criteria [11], who had visited a Health Centre in the previous 2 years, and signed the informed consent. Those individuals who, in the investigator’s opinion, were unable to read or understand the questionnaires, or had Chronic Obstructive Pulmonary Disease, were excluded [10].

Data were collected by personal interview, carried out by the Family Doctors themselves, with no specific training for this study.

The HRQoL was assessed using the EQ-5D questionnaire, where the patients themselves evaluated their state of health in severity levels by dimensions (including the 5 dimensions: mobility, personal care, daily activities, pain/discomfort, anxiety/depression) which were assessed at three levels (no problems, moderate problems, serious problems). An assessment was also made on a more general VAS scale ranging from 0 (worst state of health) to 100 (better state of health) [12].

Control of the asthma was determined using the Asthma Control Questionnaire (ACQ) [13]. Those patients who obtained an ACQ score lower than 0.75 were considered as well controlled, and those with a score ≥ 0.75 as not well controlled [10,13].

Using a questionnaire specifically designed for the study, the characteristics of the asthma disease were collected, as well as the adherence to treatment, and the socio-demographic variables, including: age, gender, place of residence (rural/urban), and educational levels (primary school, secondary school, university).

As regards smoking, they were classified into never smokers (means never having smoked habitually, no more than an occasional cigarette), ex-smokers (have been active smokers, but have not smoked for at least 6 months), or active smokers (smoke habitually every day).

Obesity was classified based on the body mass index (BMI), establishing 3 groups, normal weight (less than 25 kg/m2), overweight (between 25 and 30 kg/m2) and obese (more than 30 kg/m2).

The incidence of stressful events in the 15 days prior to completing the questionnaire was evaluated based on self-reporting by the patients, by asking them if they had suffered a stressful event in the previous 15 days, in general, without suggesting specific examples of a stressful events, with two response options, yes or no.

Treatment adherence was determined based on 3 variables: 1) level of therapeutic adherence according to the doctor; 2) frequency of forgetting the medication according to the patient; 3) importance of adherence according to the patient [10]. This last variable was determined by asking the patients about their level of agreement (from 0 = disagree completely, to 10 = agree completely) with the following sentence “Strict adherence to the medication prescribed by my doctor should improve my asthma symptoms”; and was stratified, by research team consensus, into two categories: <8 and ≥8 [10].

The severity of the asthma (intermittent, mild persistent, moderate persistent, severe persistent) was determined in accordance with the criteria in the Global Initiative for Asthma guide (GINA) 2006, based on the condition of the patient before treatment was started [11].

The presence of allergy sensitization was reported by the patients themselves. Questions on going to an Emergency unit, to their Family Doctor or any hospital admissions during the year prior to the study were included in the questionnaire.

The basic treatment was classified into four, mutually exclusive, categories: 1) patients treated with oral corticosteroids in the previous year; 2) those treated with any combination of long-acting beta-2 agonists and inhaled corticosteroids, with any other drug but without oral corticosteroids; 3) treated with inhaled corticosteroids without continually taking long-acting beta-2 agonists, although they may take beta-2 agonists on demand; 4) only beta-2 agonists [10].

### Statistical analysis

The mean, median, standard deviation, maximum and minimum values were calculated for the continuous variables, and the absolute and relative frequencies for the categorical variables.

The influence of the different variables included in the study on the 5 dimensions of the EQ-5D recoded dichotomically (without problems versus with problems) and the probability of obtaining values greater than the mean on the VAS scale were evaluated using multivariate logistic regression analysis. For the logistic regression model, all variables with a significance value of P < 0.2 in the univariate analysis were used as predictive variables in the multivariate analysis. The level of significance in the multivariate analysis was defined based on the confidence interval with an alpha error less than 0.05. The software SAS v8.2 was used. The study was approved by the Hospital Clínico San Carlos de Madrid Clinical Research Ethics Committee.

## Results

A total of 2,125 patients were finally included, with 57.7% females, and a mean age of 48 years. The response rate was 95.4%. The main characteristics of the patients included are summarized in Table 1.

**Table 1:** Factors associated with health-related quality of life (n = 2,125)

	**% patients without problems**
**Gender**	
Female	883/1,227 (72.0%)
Male	726/898 (80.8 %)
**Age**	
≥ 60 years	335/638 (52.5%)
40 to 59 years	551/704 (78.3%)
18 to 39 years	723/783 (92.3%)
**BMI (Kg/m**^**2**^**)**	
≥ 30	243/424 (57.3%)
≥ 25 to 30	656/873 (75.1%)
< 25	709/826 (85.8%)
**Educational level**	
None	74/170 (43.5%)
Primary	613/900 (68.1%)
Secondary	600/706 (85.0%)
University	319/345 (92.5%)
**Occupation**	
Non active worker	648/1,029 (63.0%)
Active worker	960/1,094 (87.7%)
**Living status**	
Lives alone	145/237 (61.2%)
Lives with someone	1461/1,885 (77.5%)
**Place of residence**	
Rural	632/859 (73.6%)
Urban	977/1,265 (77.2%)
**Family history of asthma**	
Yes	744/984 (75.6%)
No	865/1,141 (75.8%)
**Asthma severity**	
Severe persistent	28/90 (31.1%)
Moderate persistent	449/706 (63.6%)
Mild persistent	655/774 (84.6%)
Intermittent	476/554 (85.9%)
**Smoking habit**	
Never	1006/1,339 (75.1%)
Current or former	601/784 (76.7%)
**Alcohol intake**	
≤ 22.5 grams	705/940 (75.0%)
> 22.5 grams	732/944 (77.5%)
**Pets at home**	
Yes	473/626 (75.6%)
No	1,134/1,497 (75.7%)
**Allergy sensitization**	
No	871/1,201 (72.5%)
Yes	738/924 (79.9%)
**Stressful event last 15 days**	
Yes	207/307 (67.4%)
No	1,394/1,810 (77.0%)
**Adherence to treatment (physician’s point of view):**	
Very poor	31/40 (77.5%)
Poor	224/313 (71.6%)
Acceptable	624/805 (77.5%)
Good	562/743 (75.6%)
Very good	168/224 (75.0%)
**How often do you forget your medication?**	
>10 times every month	51/76 (67.1%)
6-10 times every month	163/223 (73.1%)
1-5 times every month	764/999 (76.5%)
Never	631/827 (76.3)
**Adherence is important (score, patient’s point of view):**	
< 8	388/531 (73.1%)
≥ 8	1,220/1,591 (76.7%)
**Asthma treatment**	
Oral corticosteroids	62/158 (39.2%)
LABA + IC	1,114/1,476 (75.5%)
Inhaled corticosteroids	180/209 (86.1%)
SABA or LABA	230/255 (90.2%)
**ACQ score**	
>1.50	437/792 (55.2%)
0.75-1.50	464/561 (82.7%)
<0.75	708/772 (91.7%)
**Asthma control(physician’s point of view):**	
Very poor	43/83 (51.8%)
Poor	172/322 (53.4%)
Controlled	504/721 (69.9%)
Total control	889/997 (89.2%)
**Asthma control (patient’s point of view)**	
Very poor	11/41 (26.8%)
Poor	97/191 (50.8%)
Controlled	432/666 (64.9%)
Total control	1,060/1,217 (87.1%)
**Hospital admissions**	
Yes	54/179 (30.2%)
No	1,555/1,946 (79.9%)
**Number of hospital admissions**	
≥2	10/52 (19.2%)
1	44/127 (34.6%)
0	1,555/1,946 (79.9%)
**Emergency visits**	
Yes	503/810 (62.1%)
No	1,106/1,315 (84.1%)
**Number of emergency visits**	
≥3	84/213 (39.0%)
1-2	419/596 (70.3%)
0	1,106/1,315 (84.1%)
**Family physician visits**	
No	97/112 (86.6%)
Yes	1,506/2,012 (75.1%)
**Number of family physician visits**	
0	97/112 (86.6%)
< 4	675/786 (85.9%)
≥ 4	831/1,221 (68.1%)

Through the analysis of the different dimensions evaluated with the EQ-5D questionnaire, the worst results corresponded to anxiety/depression, where 32% of asthmatics were shown to have problems. On the other hand, asthma seemed to have little effect on personal care, as only 8% mentioned problems on this aspect (Table 2).

**Table 2:** Factors associated with health-related quality of life, according dimensions of EuroQol-5D questionnaire (n = 2,125)

**A**	**% patients without problems**	Mobility Adjusted OR (95%CI)*
**Gender**		
Female	883/1,227 (72.0%)	1
Male	726/898 (80.8%)	1.49 (1.13,1.97)
**Age**		
≥ 60 years	335/638 (52.5%)	1
40 to 59 years	551/704 (78.3%)	1.96 (1.41,2.73)
18 to 39 years	723/783 (92.3%)	4.13 (2.71,6.32)
**BMI (Kg/m**^**2**^**)**		
≥ 30	243/424 (57.3%)	1
≥ 25 to 30	656/873 (75.1%)	1.64 (1.20,2.23)
< 25	709/826 (85.8%)	2.14 (1.50,3.04)
**Educational level**		
None	74/170 (43.5%)	1
Primary	613/900 (68.1%)	1.46 (0.97,2.20)
Secondary	600/706 (85.0%)	1.77 (1.10,2.85)
University	319/345 (92.5%)	3.19 (1.73,5.88)
**Occupation**		
Non active worker	648/1,029 (63.0%)	1
Active worker	960/1,094 (87.7%)	1.43 (1.04,1.97)
**Living status**		
Lives alone	145/237 (61.2%)	1
Lives with someone	1,461/1,885 (77.5%)	1.60 (1.10,2.33)
**ACQ score**		
>1.50	437/792 (55.2%)	1
0.75-1.50	464/561 (82.7%)	2.68 (1.96,3.66)
<0.75	708/772 (91.7%)	4.83 (3.37,6.92)
**Hospital admissions**		
Yes	54/179 (30.2%)	1
No	1,555/1,946 (79.9%)	2.64 (1.71,4.07)
**B**	**% patients without problems**	Self-care Adjusted OR (95%CI)*
**Age**		
≥ 60 years	335/638 (52.5%)	1
40 to 59 years	551/704 (78.3%)	2.25 (1.34,3.75)
18 to 39 years	723/783 (92.3%)	4.30 (1.93,9.54)
**Educational level**		
None	74/170 (43.5%)	1
Primary	613/900 (68.1%)	3.08 (1.89,5.03)
Secondary	600/706 (85.0%)	2.71 (1.40,5.24)
University	319/345 (92.5%)	3.21 (1.20,8.54)
**Occupation**		
Non active worker	648/1,029 (63.0%)	1
Active worker	960/1,094 (87.7%)	2.77 (1.52,5.04)
**Living status**		
Lives alone	145/237 (61.2%)	1
Lives with someone	1,461/1,885 (77.5%)	1.70 (1.03,2.80)
**Asthma severity**		
Severe persistent	28/90 (31.1%)	1
Moderate persistent	449/706 (63.6%)	1.81 (0.98,3.34)
Mild persistent	655/774 (84.6%)	3.32 (1.61,6.85)
Intermittent	476/554 (85.9%)	3.21 (1.38,7.46)
**Stressful event last 15 days**		
Yes	207/307 (67.4%)	1
No	1,394/1,810 (77.0%)	1.99 (1.22,3.26)
**Asthma treatment**		
Oral corticosteroids	62/158 (39.2%)	1
LABA + IC	1,114/1,476 (75.5%)	1.34 (0.77,2.32)
Inhaled corticosteroids	180/209 (86.1%)	4.07 (1.07,15.48)
SABA or LABA	230/255 (90.2%)	1.76 (0.58,5.33)
**ACQ score**		
>1.50	437/792 (55.2%)	1
0.75-1.50	464/561 (82.7%)	2.49 (1.43,4.36)
<0.75	708/772 (91.7%)	2.69 (1.41,5.14)
**Hospital admissions**		
Yes	54/179 (30.2%)	1
No	1,555/1,946 (79.9%)	2.52 (1.52,4.17)
**C**	**% patients without problems**	Usual activities Adjusted OR (95%CI)*
**Age**		
≥ 60 years	335/638 (52.5%)	1
40 to 59 years	551/704 (78.3%)	2.27 (1.63,3.16)
18 to 39 years	723/783 (92.3%)	2.67 (1.80,3.95)
**BMI (Kg/m**^**2**^**)**		
≥ 30	243/424 (57.3%)	1
≥ 25 to 30	656/873 (75.1%)	1.27 (0.93,1.73)
< 25	709/826 (85.8%)	1.43 (1.02,2.02)
**Educational level**		
None	74/170 (43.5%)	1
Primary	613/900 (68.1%)	1.51 (0.99,2.31)
Secondary	600/706 (85.0%)	1.86 (1.16,2.99)
University	319/345 (92.5%)	2.62 (1.49,4.60)
**Living status**		
Lives alone	145/237 (61.2%)	1
Lives with someone	1,461/1,885 (77.5%)	1.76 (1.23,2.53)
**Asthma severity**		
Severe persistent	28/90 (31.1%)	1
Moderate persistent	449/706 (63.6%)	1.75 (0.95,3.24)
Mild persistent	655/774 (84.6%)	1.97 (1.04,3.74)
Intermittent	476/554 (85.9%)	1.76 (0.90,3.45)
**Asthma treatment**		
Oral corticosteroids	62/158 (39.2%)	1
LABA + IC	1,114/1,476 (75.5%)	1.41 (0.88,2.23)
Inhaled corticosteroids	180/209 (86.1%)	2.43 (1.27,4.62)
SABA or LABA	230/255 (90.2%)	1.97 (1.05,3.71)
**ACQ score**		
>1.50	437/792 (55.2%)	1
0.75-1.50	464/561 (82.7%)	3.74 (2.79,5.09)
<0.75	708/772 (91.7%)	7.48 (5.29,10.58)
**Hospital admissions**		
Yes	54/179 (30.2%)	1
No	1,555/1,946 (79.9%)	1.75 (1.14,2.68)
**Emergency visits**		
Yes	503/810 (62.1%)	1
No	1,106/1,315 (84.1%)	1.67 (1.28,2.17)
**D**	**% patients without problems**	Pain/Discomfort Adjusted OR (95%CI)*
**Gender**		
Female	883/1,227 (72.0%)	1
Male	726/898 (80.8%)	1.55 (1.17,2.06)
**Age**		
≥ 60 years	335/638 (52.5%)	1
40 to 59 years	551/704 (78.3%)	2.32 (1.68,3.22)
18 to 39 years	723/783 (92.3%)	5.27 (3.52,7.88)
**BMI (Kg/m**^**2**^**)**		
≥ 30	243/424 (57.3%)	1
≥ 25 to 30	656/873 (75.1%)	1.36 (1.00,1.85)
< 25	709/826 (85.8%)	1.75 (1.25,2.47)
**Educational level**		
None	74/170 (43.5%)	1
Primary	613/900 (68.1%)	1.59 (1.02,2.49)
Secondary	600/706 (85.0%)	1.88 (1.14,3.09)
University	319/345 (92.5%)	2.23 (1.25,3.99)
**Occupation**		
Non active worker	648/1029 (63.0%)	1
Active worker	960/1094 (87.7%)	1.43 (1.06,1.93)
**Asthma severity**		
Severe persistent	28/90 (31.1%)	1
Moderate persistent	449/706 (63.6%)	2.24 (1.14,4.41)
Mild persistent	655/774 (84.6%)	3.35 (1.66,6.75)
Intermittent	476/554 (85.9%)	2.65 (1.28,5.48)
**Stressful event last 15 days**		
Yes	207/307 (67.4%)	1
No	1,394/1,810 (77.0%)	2.04 (1.46,2.83)
**ACQ score**		
>1.50	437/792 (55.2%)	1
0.75-1.50	464/561 (82.7%)	1.80 (1.33,2.44)
<0.75	708/772 (91.7%)	2.78 (1.99,3.88)
**Hospital admissions**		
Yes	54/179 (30.2%)	1
No	1,555/1,946 (79.9%)	2.05 (1.30,3.25)
**Emergency visits**		
Yes	503/810 (62.1%)	1
No	1,106/1,315 (84.1%)	1.37 (1.03,1.81)
**E**	**% patients without problems**	Anxiety/Depression Adjusted OR (95%CI)*
**Gender**		
Female	883/1,227 (72.0%)	1
Male	726/898 (80.8%)	1.84 (1.41,2.41)
**Age**		
≥ 60 years	335/638 (52.5%)	1
40 to 59 years	551/704 (78.3%)	1.35 (0.98,1.87)
18 to 39 years	723/783 (92.3%)	2.37 (1.62,3.47)
**Educational level**		
None	74/170 (43.5%)	1
Primary	613/900 (68.1%)	2.04 (1.33,3.12)
Secondary	600/706 (85.0%)	2.18 (1.36,3.49)
University	319/345 (92.5%)	2.55 (1.49,4.37)
**Place of residence**		
Rural	632/859 (73.6%)	1
Urban	977/1265 (77.2%)	1.28 (1.01,1.62)
**Asthma severity**		
Severe persistent	28/90 (31.1%)	1
Moderate persistent	449/706 (63.6%)	1.74 (0.96,3.15)
Mild persistent	655/774 (84.6%)	2.61 (1.40,4.87)
Intermittent	476/554 (85.9%)	2.27 (1.19,4.33)
**Stressful event last 15 days**		
Yes	207/307 (67.4%)	1
No	1,394/1,810 (77.0%)	5.57 (4.08,7.59)
**ACQ score**		
>1.50	437/792 (55.2%)	1
0.75-1.50	464/561 (82.7%)	1.36 (1.01,1.83)
<0.75	708/772 (91.7%)	1.66 (1.21,2.28)
**Hospital admissions**		
Yes	54/179 (30.2%)	1
No	1,555/1,946 (79.9%)	2.09 (1.36,3.22)
**Emergency visits**		
Yes	503/810 (62.1%)	1
No	1,106/1,315 (84.1%)	1.32 (1.02,1.72)

*Adjusted Odds Ratio (Adjusted by all the variables in the Table 1).

NS, Crude Odds Ratio non significant; BMI, Body mass index; SABA, Short**-**acting beta2 agonists; LABA, Long-acting beta2 agonists; SD, Standard Deviation; IC, Inhaled corticosteroids.

Advanced age, a lower education level, greater baseline severity of the asthma, poor control of the asthma, and the need to be admitted to hospital had a detrimental effect on at least 4 of the 5 EQ-5D dimensions (Table 2). Analyzing the quality of life according to the VAS and with the EQ-5D index, the characteristics with the most significant influence on analyzing the QoL by dimensions maintained their impact. Other factors also demonstrated an independent effect, amongst which the most significant one corresponded to patients who had some recent stressful events, or who gave little importance to adherence to treatment (Table 3).

**Table 3:** Factors associated with health-related quality of life: VAS scale (0 = worst health state, 100 = best health state); (n = 2034)

		**VAS score**	
	**N**	**Mean (SD)**	Adjusted mean differences (CI 95 %)*
**Age**			
≥ 60 years	609	62.38 (17.46)	
40 to 59 years	675	68.93 (16.66)	1.78 (−0.38,3.94)
18 to 39 years	750	76.34 (15.33)	4.99 (2.63,7.37)
**Educational level**			
None	162	58.57 (18.52)	
Primary	865	66.12 (17.18)	2.41 (−0.63,5.44)
Secondary	667	73.48 (16.02)	4.96 (1.64,8.28)
University	336	76.87 (14.92)	6.30 (2.69,9.90)
**Asthma severity**			
Severe persistent	87	46.78 (17.21)	
Moderate persistent	672	65.45 (16.86)	9.01 (5.07,12.96)
Mild persistent	754	72.97 (15.12)	11.40 (7.28,15.52)
Intermittent	520	74.26 (16.96)	9.49 (5.22,13.76)
**Smoking habit**			
Current or former	753	68.47 (17.02)	
Never	1,279	70.43 (17.58)	2.51 (1.13,3.89)
**Stressful event last 15 days**			
Yes	296	61.47 (18.61)	
No	1,731	71.10 (16.75)	6.17 (4.31,8.03)
**Adherence is important (score, patient’s point of view):**			
< 8	511	65.25 (17.78)	
≥ 8	1,521	71.23 (16.98)	4.02 (2.54,5.50)
**ACQ score**			
>1.50	762	58.94 (16.38)	
0.75-1.50	541	72.59 (13.89)	9.48 (7.53,11.44)
<0.75	731	78.79 (14.48)	13.26 (11.22,15.29)

Score of VAS scale, and probability of obtaining values greater than the mean on the VAS. Multivariate analysis.

*Adjusted by all the variables in the Table 1.

N, number of cases; VAS, Visual Analogue Scale; CI, Confidence interval; BMI, Body mass index; SABA, Short**-**acting beta2 agonists; LABA, Long-acting beta2 agonists; IC, Inhaled corticosteroids.

## Discussion

It appears to be a current need to assess HRQoL in asthma, although it is still not clear which is the most suitable tool for assessing it [14]. EQ-5D is a generic HRQoL questionnaire that correlates well with asthma specific questionnaires. Also, the broader approach of generic questionnaires can give them a higher potential to pick up unexpected aspects or collateral effects of the disease. These two factors make it reasonably valid for population studies with asthmatic patients [5,15-17]. Furthermore, being a generic questionnaire, it helps in making the cost comparison with other chronic illnesses [14]. The response rate obtained in our study was relatively high (95.4%), and probably associated, at least partly, with the simplicity and rapidity in completing the EQ-5D questionnaire; which also makes it easier to use in daily practice [18].

Our results appear to confirm the negative effect of asthma on HRQoL when assessed with the EQ-5D questionnaire, and this effect appears worse than that reported in the general Spanish population [19]. Only the pain/discomfort dimension and the values obtained on the VAS for the general population are similar to those of our asthmatic population, but there are significant differences in the rest of the dimensions [19].

Increasing age, lower educational level, poorer asthma control and requiring hospital admissions were significant determining factors of a poorer HRQoL in asthmatic patients in all the dimensions analyzed, as well as in the general HRQoL analyzed using the VAS.

Although in some studies with few patients [20], or with a relatively young population [21] no association was seen between age and HRQoL, the majority of authors mention a deterioration in HRQoL with increasing age [5,8,22]. Some factors associated with ageing could explain this result. On the one hand, there are changes in lung function, such as an increase in airway hyper-responsiveness, accelerated decline in forced expiratory volume in one second, higher prevalence of irreversible airway obstruction, increased air trapping, reduction in chest wall compliance, a decrease in static elastic recoil pressure of the lung, reduction in respiratory muscle strength, as well as changes in chest configuration which make respiratory movements difficult [23,24]. There are also changes in the immune system, immunosenescence, which increases susceptibility to infections and malignancy rates [25]. Likewise, a deterioration in sight and hearing, in motor abilities, or even incipient cognitive impairment, can make adherence to treatment difficult [23]. Finally, comorbidity is more frequent in advanced age, as such that it may increase the symptomatology, and even make it difficult to indicate some treatments that may be suitable for asthma, but contraindicated by some of the respiratory comorbidities [23,24].

Lower health literacy has been reported in patients with a lower education level, as well as, lower mathematical skills, more delayed diagnosis of asthma, poorer access to health care or less adherence to healthy lifestyles, which could contribute to the worsening of HRQoL observed in these patients [26-29].

Hospital admissions are a major predictor of a worse HRQoL in asthma [8]; but a worse HRQoL is also associated with a higher probability of hospital admissions [30].

Good control of the disease has a significant effect on a better HRQoL, which is in agreement with other authors [5,31]. However, to achieve good control of the disease does not imply obtaining an optimum HRQoL, as other factors, such as the presence of the disease itself, or the need for treatment and medical care, although improving control of the asthma, have an effect on the HRQoL [31].

Although there is a known relationship between asthma and psychological factors, the impact of psychological distress on the health of asthmatics and its determining factors are not well described [32]. Some authors observed a worse quality of life in asthmatics with anxiety and/or depression [33-35]. On the other hand, Heaney et al., observed that the improvement in asthma control led to a better quality of life, but did not change the psychiatric symptoms. [36]. Furthermore, there is no single method to assess negative life events (NLE), understood as events that occur in the patient’s life that lead to stress, such as financial losses, work difficulties, death of a close friend or family, or other family problems [3]. In our case, this information was provided by the patients themselves. Behavioural and pathophysiological changes have been reported as determining factors in these NLE on asthma. For the former aspect, self-care could be reduced, or inadequate use of the medication [32,37], and for the latter, an increase in airway inflammation has recently been demonstrated in asthmatic patients after stressful events [38].

Patients with more severe forms of asthma have a worse HRQoL, both in our population and in others [8,39]. However, a recent population study in the USA did not see a relationship between severity and the HRQoL [5]. Another study in a Japanese population, observed deterioration in the HRQoL with the increasing severity of asthma in males, but not in females, establishing the possibility of different “coping styles” between the sexes [40].

Patients who remain in active employment have a better QoL, a similar finding to that of Siroux in a multicentre European study [8]. Lack of occupational activity is associated with a lower socio-economic income and greater psychological distress, which could be determining factors in the deterioration of the QoL [3,41].

Living in an urban setting is associated with a better HRQoL. The same relationship was observed in a large study with Spanish children, suggesting the possibility that urban areas could have better access to the health system [42]. Other differences have also been identified in the management of the disease, with a lower likelihood of scheduled follow up or a greater under-diagnosis in patients in rural areas [43,44].

The damaging effect of living alone appears to be confirmed, given that it is associated to a worse QoL. This is in agreement with other studies, where to live alone was a predictive factor of more visits to the Emergency Department, worse mental health or longer delay in the diagnosis [29,45,46].

In other aspects analyzed, our findings agree with those mentioned in the literature, showing more exacerbations [8,30]; female sex [8,47]; less adherence to treatment [48]; being someone who never smoked [5,49]; and obesity [8,50] are determining factors of a worse quality of life.

Our study may have some limitations. Firstly, being a cross-sectional study causality relationships cannot be defined. Secondly, the reference NLE is by self-report, as such any influence of the personality of the patients cannot be excluded. Thirdly, the selection of the participating doctors was not strictly randomized. However, given the high number of participating doctors and the distribution over the whole country, as well as the collection of data with validated tools like the EQ-5D questionnaire, the inclusion of a significant number of co-variables that could have an influence on the quality of life, and the high number of patients analyzed, these results should be reasonably valid as representative of the reality of the QoL of asthmatics in Spain and its determining factors.

## Conclusions

In our study, we could identify some factors related to worst quality of life in asthma patients.

The most significant were advanced age, lower education level, greater baseline severity of the asthma, presence of stressful events, poor control of the asthma, and need to be admitted to hospital.

We believe that the identification of factors related to poor asthma control, and particularly those that could be changed by the health care system [10], could lead to an improvement in the situation of asthmatic patients. Thus, the prevention and treatment of obesity and smoking, as well as reinforcing health education, seem to be basic aspects that could be performed by the health care system.

## Abbreviations

HRQoL: Health-related quality of life; EQ-5D: EuroQol-5D; VAS: Visual analogue scale; BMI: Body mass index; GINA: Global Initiative for Asthma guide.

## Competing interests

Mónica Tafalla and Javier Nuevo are employees of AstraZeneca Spain. The study was funded by AstraZeneca Spain.

## Authors' contributions

Designed research/study: Mónica Tafalla, Javier Nuevo, Francisco Caamaño-Isorna. Performed research/study: Mónica Tafalla, Javier Nuevo, Francisco Caamaño-Isorna. Collected data: Mónica Tafalla, Javier Nuevo, Francisco Caamaño-Isorna. Analyzed data: Francisco-Javier Gonzalez-Barcala, Ramon de la Fuente-Cid, MónicaTafalla, Javier Nuevo, Francisco Caamaño-Isorna. Wrote paper: Francisco-Javier Gonzalez-Barcala, Ramon de la Fuente-Cid. All authors read and approved the final manuscript.
